# Long-term recurrent in situ melanoma

**DOI:** 10.1016/j.jdcr.2026.03.067

**Published:** 2026-04-21

**Authors:** Jonathan Stevens, Nadia Vega, Catalina Retamal, Cecilia Jeraldo

**Affiliations:** a Dermatology Service, Hospital del Salvador, Santiago, Chile; b Dermatology Department, Institute of Oncology, Foundation Arturo Lopez Perez (FALP), Santiago, Chile; c Department of Dermatology, Faculty of Medicine, University of Chile, Santiago, Chile; d Pathology Service, Hospital del Salvador, Santiago, Chile

**Keywords:** dermoscopy, recurrent melanoma, scar, skin neoplasms

## Clinical presentation

**Fig 1:**
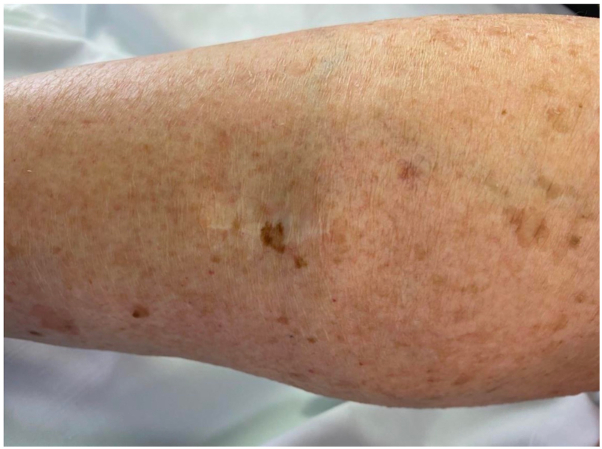
Clinical image. A scar with an asymmetrical, hyperpigmented 7 × 10 mm macule extending beyond the limits of the scar. Adjacent to the scar, there is a 3-mm asymmetrical brown macule.

A 74-year-old woman with a history of an atypical melanocytic lesion on her left leg underwent excisional biopsy, which revealed melanoma in situ. Histopathology showed clear margins, with 2 mm lateral and deep clearance. A wide excision was not performed at the time. Twelve years later, she presented with 2 new melanocytic lesions on the scar, which had developed over the past 3 months (Fig 1).

## Dermoscopic appearance

**Fig 2:**
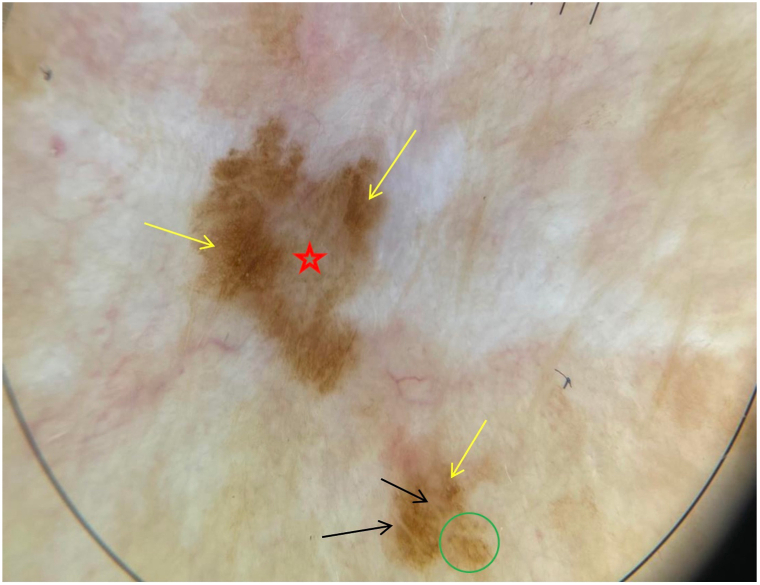
Dermoscopy of the lesion. Dermatoscopy showed a scar with a hyperpigmented macule, revealing an atypical, thickened pigment network (*yellow arrows*) and a central unstructured area (*red star*). The adjacent brown macule displays an atypical pigment network (*yellow arrow*), marked skin lines (*black arrows*), and eccentric globules at the periphery (*green circle*).

Dermoscopy revealed an atypical pigment network, structureless areas, and eccentric globules extending beyond the scar’s edges (Fig 2).

## Histologic diagnosis

**Fig 3:**
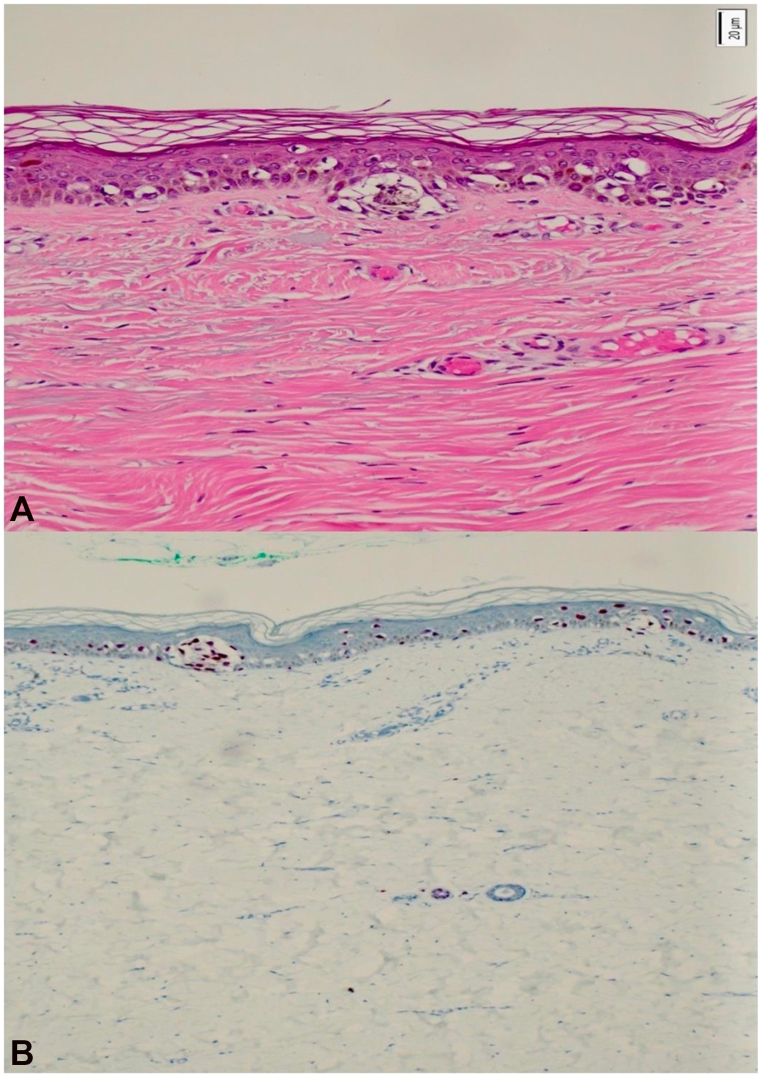
**A,** Histopathology examination showing recurrent intraepidermal malignant melanoma, with a lateral margin in contact with the main lesion, and no evidence of vascular or neural permeation. **B,** Immunohistochemical shows atypical melanocytes tested positive for Sox-10 staining.

Both lesions were excised. Histopathologic examination confirmed a recurrent in situ melanoma (Fig 3, *A*).

A subsequent wide excision with a 1-cm margin extending to the muscular fascia was performed, and histopathology showed no residual neoplasia. After 24 months of follow up, there was no clinical evidence of recurrence.

Key message

Local recurrence of melanoma is defined as regrowth within 2 cm of the surgical scar after primary excision, most commonly as a result of inadequate excision of the original tumor.^1^ When excision is performed with a 5-mm surgical margin, melanoma in situ has been reported to have a low recurrence rate of 0.9%.^2^ Recurrent melanoma is strongly associated with specific clinical and dermoscopic features.^3^ In our case, the prolonged interval since excision, along with pigmentation extending beyond the scar, a noncontinuous growth pattern, an atypical pigment network, and peripheral eccentric globules were all suggestive of recurrence. Dermoscopy plays a key role in enabling timely diagnosis, as recurrent melanoma typically exhibits characteristic dermoscopic findings, including chaotic and discontinuous growth pattern, eccentric hyperpigmentation, and pigmentation extending beyond the edges of the scar.^3^

## Conflicts of interest

None disclosed.
